# Safety of non-woven polypropylene surgical adhesive drapes to prevent wound infection

**DOI:** 10.1186/1753-6561-5-S6-P190

**Published:** 2011-06-29

**Authors:** A Jeurissen, R Hendrickx, K Beesemans, A Van Thielen, V Cossey, A Schuermans

**Affiliations:** 1 1Hospital hygiene and infection control, UzZ Gasthuiberg, Leuven, Belgium

## Introduction / objectives

Wound infections caused by intra-operative contamination can be a major problem in surgery. The passage of bacteria through drapes is a potential source of wound infection. In this study we aimed to test the bacterial penetrability of 6 brands of non-woven polypropylene drapes.

## Methods

Six brands of disposable non-woven polypropylene drapes were tested. A latex glove was used as a negative control and a woven cotton drape was used as a positive control. For each drape, a rodac plate was inoculated with 10^5^ colony-forming units / ml of *Staphylococcus aureus* ATCC strain 6538 and incubated at 37°C for 24 h to obtain confluent growth. Thereafter, each drape was placed between the incubated rodac plate and an inverted rodac plate with a weight of 400 gram placed thereon for 30 minutes. Subsequently, the inverted rodac plate was incubated for 24 h at 37°C and inspected for growth of *S. aureus*.

## Results

The latex glove was totally impermeable, in contrast to the woven cotton drape which revealed heavy growth after 30 minutes. All the drapes made from non-woven polypropylene were impermeable.

## Conclusion

Although bacterial penetration through surgical drapes can be time dependent, we here show that polypropylene non-woven drapes were impenetrable at 30 minutes. We therefore recommend the use of non-woven polypropylene drapes in surgical procedures

## Disclosure of interest

None declared.

